# Expression of ionotropic receptors in terrestrial hermit crab's olfactory sensory neurons

**DOI:** 10.3389/fncel.2014.00448

**Published:** 2015-02-02

**Authors:** Katrin C. Groh-Lunow, Merid N. Getahun, Ewald Grosse-Wilde, Bill S. Hansson

**Affiliations:** Department of Evolutionary Neuroethology, Max Planck Institute for Chemical EcologyJena, Germany

**Keywords:** crustacea, antennules, olfaction, ionotropic receptors, *in situ* hybridization, electrophysiology

## Abstract

Coenobitidae are one out of at least five crustacean lineages which independently succeeded in the transition from water to land. This change in lifestyle required adaptation of the peripheral olfactory organs, the antennules, in order to sense chemical cues in the new terrestrial habitat. Hermit crab olfactory aesthetascs are arranged in a field on the distal segment of the antennular flagellum. Aesthetascs house approximately 300 dendrites with their cell bodies arranged in spindle-like complexes of ca. 150 cell bodies each. While the aesthetascs of aquatic crustaceans have been shown to be the place of odor uptake and previous studies identified ionotropic receptors (IRs) as the putative chemosensory receptors expressed in decapod antennules, the expression of IRs besides the IR co-receptors IR25a and IR93a in olfactory sensory neurons (OSNs) has not been documented yet. Our goal was to reveal the expression and distribution pattern of non-co-receptor IRs in OSNs of *Coenobita clypeatus*, a terrestrial hermit crab, with RNA *in situ* hybridization. We expanded our previously published RNAseq dataset, and revealed 22 novel IR candidates in the *Coenobita* antennules. We then used RNA probes directed against three different IRs to visualize their expression within the OSN cell body complexes. Furthermore we aimed to characterize ligand spectra of single aesthetascs by recording local field potentials and responses from individual dendrites. This also allowed comparison to functional data from insect OSNs expressing antennal IRs. We show that this orphan receptor subgroup with presumably non-olfactory function in insects is likely the basis of olfaction in terrestrial hermit crabs.

## Introduction

One of the biggest steps in animal evolution was the successful transition from water to land. Among arthropods, this step was successfully and independently accomplished by several taxa, including at least five distinct crustacean lineages (Bliss and Mantel, 1968; Powers and Bliss, 1983). One of these are the hermit crabs (Coenobitidae), who transitioned approximately 20 mya according to fossil records (Glaessner, 1969). Species with a predominantly or exclusively terrestrial lifestyle include *Birgus latro*, which forms its own genus, and the genus *Coenobita* with approximately 15 extant species. Substantial changes were required to adapt to the new environment in terms of water and ion balance, metabolism and modification of sensory organs in order to receive stimuli from a changed conveying medium (Greenaway, 2003). The crustacean antennae are important sensory organs, with the second pair of antennae being primarily mechanosensors, while the first pair of antennae, also called antennules, are olfactory organs (Eder and Atema, 1978; Koehl et al., 2001). Hermit crabs are known to rely on their chemical sense in many contexts, including predator avoidance and in search of food, fresh and salt water and resources like empty snail shells to protect their soft abdomen (Small and Thacker, 1994; Thacker, 1997; Rosen et al., 2009; Krång et al., 2012).

The crustacean sister phylum, the insects, succeeded in the transition from water to land approximately 600 mya (Richter et al., 2009; Little, 2009). In the course of evolution, insects employed two different chemoreceptor classes: the Olfactory Receptors (ORs), a gene family most likely derived from the older Gustatory Receptors (GRs), and the antennal Ionotropic Receptors (IRs) (Robertson et al., 2003; Benton et al., 2009). GRs but no ORs are present in the genome of *Daphnia pulex*, the only complete crustacean genome available to date (Penalva-Arana et al., 2009). ORs appeared in the ordovician insect lineage, around the time of Zygentoma (Missbach et al., 2014). IRs, however, are derived from the ancient family of ionotropic glutamate receptors, with the earliest occurrence of one of the antennal IR co-receptor, IR25a, in the early protostomian lineage, more than 600 Mya (Croset et al., 2010; Rota-Stabelli et al., 2013). Insect IRs are subdivided into two groups based on their expression in *Drosophila melanogaster*: the “antennal IRs,” expressed in sensilla of the antennae, and “divergent IRs,” which are not antennaly expressed and have not been functionally characterized yet (Benton et al., 2009; Croset et al., 2010; Silbering et al., 2011). The *Daphnia* genome yielded a large set of GRs, two antennal IRs, the ancient co-receptors IR25a and IR93a, and a high number of divergent IRs (Croset et al., 2010). The co-receptor IR25a is expressed in all OSN cell bodies of the American lobster *Homarus americanus* and the spiny lobster *Panulirus argus* (Hollins et al., 2003; Corey et al., 2013). The latter also expresses IR93a as well as two divergent IRs in its antennules (Corey et al., 2013). In terrestrial hermit crabs, IR25a, IR93a and seven divergent IRs were identified from the antennal transcriptome of *C. clypeatus* (Groh et al., 2014). IRs are the only putative chemoreceptors identified in the crustacean antennules so far, as GRs are not expressed in this tissue (Stepanyan et al., 2006; Corey et al., 2013; Groh et al., 2014). However, it remains to be investigated if the non-co-receptor IRs identified from transcriptomic data are expressed in olfactory sensory neurons (OSNs). OSN cell bodies are situated below so-called aesthetascs, cuticular structures comparable to insect sensilla arranged in rows on the last antennular segment and housing the OSN dendrites (Ghiradella et al., 1968b; Gleeson, 1982). While in aquatic decapods the aesthetascs are long and slender with OSN cell body clusters lying shallow underneath the cuticle, the aesthetascs of *C. clypeatus* are short and blunt, with OSN cell bodies arranged in spindle like complexes of approximately 150 cell bodies each, which are withdrawn deeper into the last antennular segment (Ghiradella et al., 1968a; Stensmyr et al., 2005; Hansson et al., 2011; Koczan, 2012).

Based on a collection of odors that were tested in previous studies (Stensmyr et al., 2005; Krång et al., 2012) we tested the responsiveness of single aesthetascs and individual dendrites aiming to characterize their ligand spectra. Expanding on our previous investigation of the antennal transcriptome of *C. clypeatus*, we generated a dataset with more depth for a more complete assessment of the IR repertoire and cloned several receptors. Finally we employed fluorescent RNA *in situ* hybridization with probes targeting these receptors to demonstrate expression of divergent IRs in OSN cell bodies.

## Materials and methods

### Animals and sample preparation

Specimens of *Coenobita clypeatus* were ordered from Peter Hoch Import—Export Waldkirch (Germany). *C. clypeatus* is a species neither endangered nor protected. Cold anesthetization of specimen was followed by antennulae dissection by scissors. Dissected antennules were either pooled for RNA extraction or transferred into fixation buffer for *in situ* experiments. All experiments were carried out in accordance with the national ethical guidelines (“genehmigungsfreien Versuchsvorhabens nach § 8a Abs.1 und 2 des Tierschutzgesetzes Deutschland vom 18. Mai, 2006 BGBl. I S. 1206”), including notification and consent of the responsible administrative authorities responsible for the Max Planck Institute for Chemical Ecology in Jena.

### RNA preparation and RNAseq

Antennules of 10 specimens were homogenized in a Tissue-Lyzer (Invitrogen) in 1 ml TRIreagent (Sigma) at 50 hz for 3 min. The following steps were carried out according to the manufacturer's instructions, replacing chloroform with 1-bromo-3-chloro-propane. Total RNA preparations were sent to the Max Planck-Genome-Centre Cologne for TruSeq RNA sequencing resulting in 29,987,467 reads. Raw sequencing data is available for download (EBI/ENA Study acc.number ERP005273). Data was screened for contaminants and linker-/adapter sequences followed by *de novo* assembly of the sanitized reads in CLC genomics workbench V 6.

### Bioinformatics

A BLAST2GO database was created based on the contigs with homology searches after dynamic translation (BLASTX) against non-redundant databases (National Center for Biotechnology Information, NCBI) using the default cutoff parameters (1.0E-3) and InterProScan (Conesa et al., 2005). GO graphs were calculated with a cutoff set to 10. All contigs were included in BLAST searches of selected genes of interest. Dendrograms were compiled using the MUSCLE multiple sequence alignment tool (Edgar, 2004) followed by FastTree dendrogram calculation (Price et al., 2010). Adobe Illustrator CS5 was employed to compile figures.

The data is available for download here:

- Sequencing data EBI/ENA Study accession number ERP005273
- Sequences of annotated transcripts: ENA: http://www.ebi.ac.uk/ena/data/view/LN590512-LN590533

### Probe synthesis for *in situ* hybridization

Total RNA preparations from 35 pairs of antennules were carried out as described for RNAseq above. RNA solution was cleaned up using the Poly(A)Purist™ MAG Kit (Ambion, Kaufungen, Germany) according to the manufacturer's instructions. Clean poly-A-RNA was transcribed to cDNA using SMARTer™ RACE cDNA Amplification Kit (Takara Bio Europe/Clontech, Saint-Germain-en-Laye, France) generating both 5′RACE-Ready and 3′RACE-Ready cDNA. Based on the contigs from *de novo* assembled RNAseq data primers were designed using Primer3 (http://bioinfo.ut.ee/primer3-0.4.0/) and reviewed using Oligo Calc (http://www.basic.northwestern.edu/biotools/OligoCalc.html). PCR was carried out using Advantage® 2 PCR kit (Takara Bio Europe/Clontech, Saint-Germain-en-Laye, France) according to manufacturer's instructions. Obtained PCR fragments were cloned and sequenced. Clones matching the sequence of the respective contig were selected and linearized following recommended protocols for generating *in situ* hybridization probes using the SP6/T7 RNA transcription system with DIG (digoxygenin) labeling (Roche Diagnostics, Risch, Switzerland). Probes were subsequently shortened to 600-800 nucleotides length using a carbonate buffer (80 mM NaHCO_3_, 120 mM Na_2_CO_3_, pH 10.2) following the protocol of (Angerer and Angerer, 1992). All probes were synthesized in sense- and antisense direction and used in parallel in all *in situ* experiments.

### *In situ* hybridization

Antennules were cut as described above and transferred to 4% paraformaldehyde in 0.1 M NaCO_3_, pH 9.5 for 24 h. Fixed antennules were individually dissected in PBS (phosphate-buffered saline = 0.85% NaCl, 1.4 mM KH_2_PO_4_, 8 mM Na_2_ HPO_4_, pH 7.1, 0.03% Triton X 100) removing one side of the lateral cuticle. Afterwards we proceeded as described by Schymura et al. (2010), replacing WM-HBL by Hybridization Buffer (50% formamide, 2× SSC, 10% dextran sulfate, 20 mg/ml yeast t-RNA, 0.2 mg/ml herring sperm DNA) due to better performance and DAP buffer by Detection Buffer (0.1 M TRIS pH 9.5, 0.1 M NaCl, 0.01 M MgCl_2_, pH 8) according to manufacturer's instructions to the HNPP Fluorescent Detection Set (Roche Diagnostics, Risch, Switzerland). Antennules were subsequently stained 10 min in SYTOX blue solution (1:2000 in PBS) and washed 3 times 10 min with PBS. After the last washing, preparations were mounted on glass slides in PBS-Glycerol (1:3), covered with cover-slips and sealed with nail polish. Signals were visualized using an LSM 510 Meta confocal microscope (Zeiss, Jena, Germany) and Zeiss image browser. Settings for laser intensity and detection were not changed between scanning samples with sense and antisense probes of the same IR.

### Statistics

*In situ* signals of four to six spindle-like complexes of OSNs per antennular region in antennules of at least two independent runs were counted. The regions were defined as “base” (proximal third of the last antennular section), “mid” (medial third of the last antennular section) and “tip” (distal third of the last antennular section). Regions were compared in a One-Way ANOVA using R studio version 2.12.2 (2011-02-25) (Copyright © 2011 The R Foundation for Statistical Computing).

### Immunohistochemistry

Antennules were cut and prepared as described above. After treatment with HCl as described in Schymura et al. (2010) preparations were washed in PBS, blocked overnight in blocking solution (PBS with 2% NGS, 0.3% Triton X 100, 0.05% Na-azide), incubated shaking for 6 h at room temperature and 2 days at 8°C with primary antibody against IR25a kindly provided by Elizabeth Corey (Stepanyan et al., 2004; Corey et al., 2013). After washing in PBS the secondary antibody (Goat anti rabbit coupled to fluorophore Alexa 488) was applied and incubated at 8 degrees Celsius overnight. Preparations were counterstained with SYTOX orange, washed, mounted and visualized as described above. For control, preparations were incubated without primary antibody.

### Electrophysiology

In order to measure electrical activity of OSNs in hermit crab aesthetascs, whole animals were mounted in 15 ml falcon tubes after shell removal. Moistened tissue paper was used to prevent back movement and to keep the animals moist. A humidified air flow was continuously directed on the antennulae at the rate of 2 l/min during the recording period. Extracellular recording was performed by inserting a glass electrode filled with sensillum lymph ringer solution (Kaissling and Thorson, 1980) at the base of aesthetasc. To see if physiological responses between individual aesthetascs and between the regions were different, electrophysiological recordings were taken from aesthetascs in the three regions (proximal, medial and distal, see **Figure 4**). Both a change in spike rate from single aesthetascs and a deflection in the local field potential (LFP) which, is a measure of dendrites current (Schneider, 1969; Kaissling, 1986; Nagel and Wilson, 2011) were recorded after odor stimulation of antennules. For grounding, silver wire was inserted into the thorax behind the eyestalks. 21 odors were selected from a set previously tested for activity in electroantennographic recordings of hermit crabs to characterize the functionality of aesthetasc OSNs (Stensmyr et al., 2005; Krång et al., 2012). Odors were also selected to represent key compounds of hermit crab food sources, such as isoamyl acetate of banana or ethyl hexanoate of apple (Stensmyr et al., 2005; Krång et al., 2012), and the Henkel 100 mixture (kindly provided by Dr. Thomas Gerke, Henkel, Germany) was chosen as a broad general odor mixture. All odors except Henkel 100 were dissolved in water at the dilution of 10-1 v/v, Henkel 100 in hexane. Ammonium hydroxide solution is 28% NH3 in water. For each region of the antenna at least five animals were tested.

## Results

### Sequencing and assembly

RNAseq based transcriptomes allow access to expression information of genes. A high read count results in a so called deep dataset, representing expression of most or all genes of a given tissue sample. Here, Solexa/Illumina sequencing of antennular RNA generated 29,987,467 sequencing reads of 96 nucleotides length each. Assembly by CLC genomics workbench 6, after Vector/Adapter scan and cleanup, resulted in a total of 73,235 contigs above 200 nucleotides length with an N50 of 744 bases. All contigs were included in the subsequent analysis.

### GO annotation

**Figure 1:**
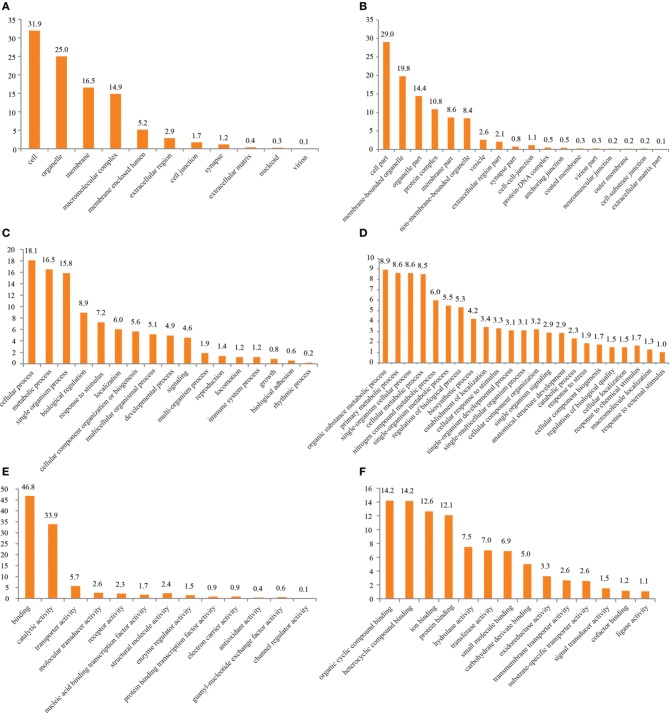
**GO term distribution after annotation by BLAST2GO. (A)** Cellular component level 2, **(B)** cellular component level 3, **(C)** molecular function level 2, **(D)** molecular function level 3, **(E)** biological Process level 2, **(F)** biological process level 3.

Access to the complexity of a given tissue is made easier by applying a generalized vocabulary, connecting knowledge of processes and functions of particular molecules to a transferable hierarchy of terms. Such a vocabulary is provided by GO classification (Ashburner et al., 2000) and was applied to the data using BLAST2GO as described in Conesa et al. (2005). 18,583 out of 73,235 contigs (25.37%) yielded a BLAST result after dynamic translation (BLASTX) while InterProScan identified known domains in 28,593 contigs (39.04%). This confirms our earlier investigations of the same tissue (Groh et al., 2014), extending the total number of transcripts but pointing out the lack of identified orthologs and functional data for their majority. Figure 1 surveys the general distribution of assigned terms on the second and third level of the three categories “Cellular Component,” “Molecular Function,” and “Biological Process” (full list of terms and proportions in Supplementary Table 1). Comparable to literature data of decapod antennules and functionally similar tissues (Grosse-Wilde et al., 2011; Legeai et al., 2011; Corey et al., 2013; Groh et al., 2014), most of the contigs were assigned to the term “cell” (44%) followed by “organelle” (29%) in the category of “Cellular Component” on 2nd level (Figure 1A). On level 3, the most abundant term was “cell part” (39%) followed by “membrane bounded organelle” (22%) (Figure 1B). Term distribution in the category of “Molecular Function” largely confirmed the trend of term distribution found in our previous study (Groh et al., 2014) but varied to a small extent in the proportions. Most ESTs on level 2 were assigned to terms of enzymatic functions, like “binding” (46%) and “catalytic activity” (36%) (Figure 1C). On level 3 a total of 44 terms was assigned to the data. Figure 1D therefore depicts only terms with an abundance of at least 0.5% of total terms on that level, easing visibility. A full list of terms is given in Supplementary Table 1. The highest proportion of ESTs was found in the term “protein binding” (15%), followed by “hydrolase activity” (11%) and “nucleotide binding” (10%). Expectedly, the highest proportion of ESTs on level 2 “Biological Process” is assigned to cellular processes (24%) and metabolism (20%) in general (Figure 1E). As the antennules' main function is chemoreception, terms associated to sensory tasks like “signaling” (5%) and “response to stimulus” (5%) were present as described in sensory tissues of other arthropods Grosse-Wilde et al., 2011; Andersson et al., 2013; Cao et al., 2014. A total of 92 terms was assigned to the data in the category “Biological Process” on level 3. Again only terms with an abundance of at least 0.5% of total terms on that level are included in the depiction in Figure 1F. The highest proportion of terms included ESTs involved in basic processes of metabolism and its regulation, but also more sensory specific processes, like “signaling process” (2%) and “signaling pathway” (2%), as well as “response to abiotic stimulus” (0.7%).

### Olfactory pathway

Studies in lobster indicate an involvement of G-protein coupled receptor (GPCR) signaling in peripheral signal processing (Corey et al., 2010; Bobkov et al., 2012). Supplementary Table 2 displays a list of contigs connected to the GO term “GPCR signaling” with their respective similarity to database entry homologs. The identified contigs include GPCRs, subunits of the GPCR signaling cascade as well as regulators of this signaling process.

### Identification of IRs

**Figure 2:**
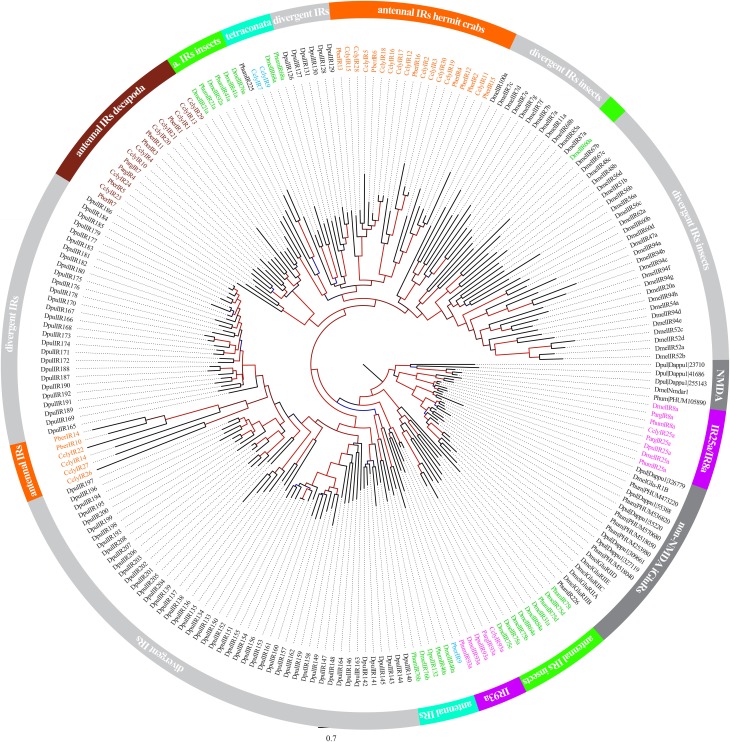
**Analysis of Ionotropic Receptor relationships between *C. clypeatus, P. bernhardus, P. argus, D. pulex, P. humanus humanus* and *D. melanogaster***. (Sequences of *P. bernhardus* from Groh et al., 2014; and *C. clypeatus* from this study and Groh et al., 2014; *P. argus* from Corey et al., 2013; *D. pulex, P. humanus*, and *D. melanogaster* from Croset et al., 2010). Alignment of aminoacid sequences using muscle (Edgar, 2004); dendrogram compilation by FastTree (Price et al., 2010); support values of branches indicated by color gradient (blue to black 0–0.5, black to red 0.5–1).

Recent investigations of decapod antennulae indicated the ionotropic receptors (IRs) to be the only chemosensory receptors present in antennules (Stepanyan et al., 2006; Corey et al., 2013; Groh et al., 2014). We used publicly available IR sequences to screen our RNAseq-based dataset for homologs, revealing 46 candidate sequences, including the previously identified CclyIR1, IR2, IR3, IR4, IR5, IR93a, and IR25a. By using RACE-PCR contigs were verified and extended toward both ends while only IR93a was successfully extended this way to full length. Nevertheless, 22 of the novel IR candidate sequences were spanning at least two of the three characteristic transmembrane domains of IRs and could therefore be considered as unigenes (sequences available from ENA: http://www.ebi.ac.uk/ena/data/view/LN590512-LN590533). The dendrogram depicted in Figure 2 is based on aminoacid multiple sequence alignments of our 29 known and novel candidate IRs and reference sequences of IRs retrieved from NCBI, including *Daphnia pulex, Panulirus argus, Pagurus bernhardus, Pediculus humanus humanus*, and *Drosophila melanogaster*. The analysis included all described IRs of crustaceans. For higher reliability only the region including the ion channel pore until the 3-prime end of the sequences was included, similar to the method described in Croset et al. (2010). Besides the IR-co-receptors IR25a and IR93a, both known to be broadly and exclusively expressed in lobster OSN populations (Hollins et al., 2003; Corey et al., 2013), most IRs of *C. clypeatus* formed distinct clusters together with IRs from other decapods and apart from the antennal IRs of *D. melanogaster*. The largest cluster consisted of 13 CclyIR candidates, 7 IRs of *P. bernhardus* and both known non-co-receptor IRs of *P. argus*. The second largest cluster, composed of 10 CclyIR and 4 PberIR candidates, was neighboring a group consisting of two CclyIRs and the antennal DmelIR68a. Another 2 Ccly and 4PberIR candidates were next to a cluster of *D. pulex* divergent IRs.

### Expression of IRs in OSNs

**Figure 3:**
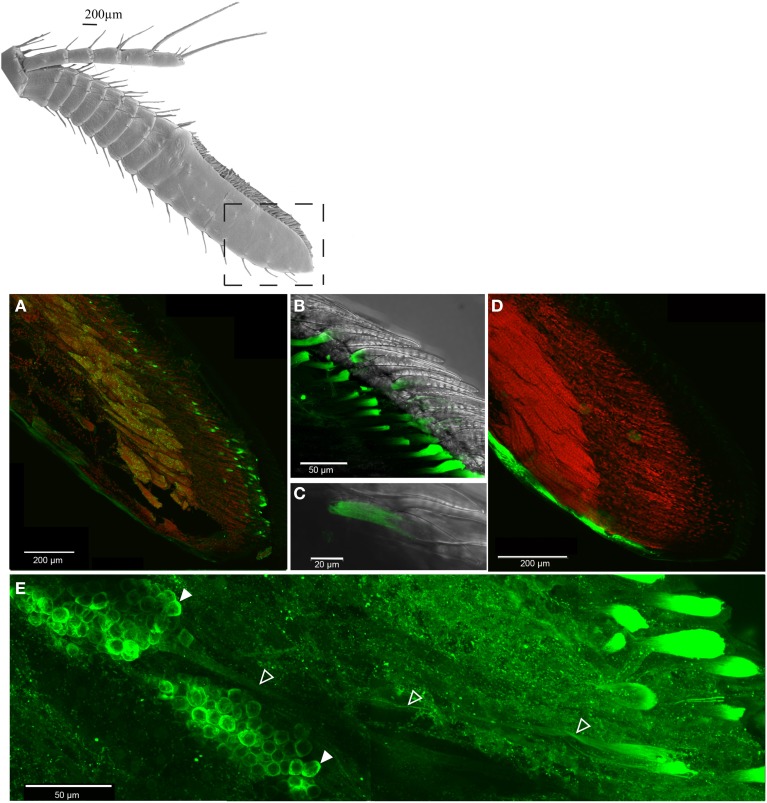
**Distribution of IR co-receptor IR25a in the last antennular section, LSM scan of whole mount immunohistochemical assay, IR25a protein labeled in green, counterstain of cell core with SYTOX orange, **(A)** overview of OSN cell bodies expressing IR25a, **(B)** IR25a in the aesthetasc dendrites, **(C)** closeup of an exemplary aesthetasc, **(D)** control without primary antibody, **(E)** IR25a in dendrites (outlined arrowheads) from cell bodies (filled arrowheads) to the aesthetasc pad**.

Immunohistochemical experiments revealed expression of the IR co-receptor IR25a in virtually all OSN cell bodies of the antennules (Figure 3A). Dendrites were continuously labeled from the spindle-like OSN cell body complexes to the aesthetasc patch (Figure 3E), and especially both in close proximity and inside the aesthetasc cuticle (Figures 3B,C). Control experiments without primary antibody application showed no labeling of any cells in the antennules (Figure 3D).

**Figure 4:**
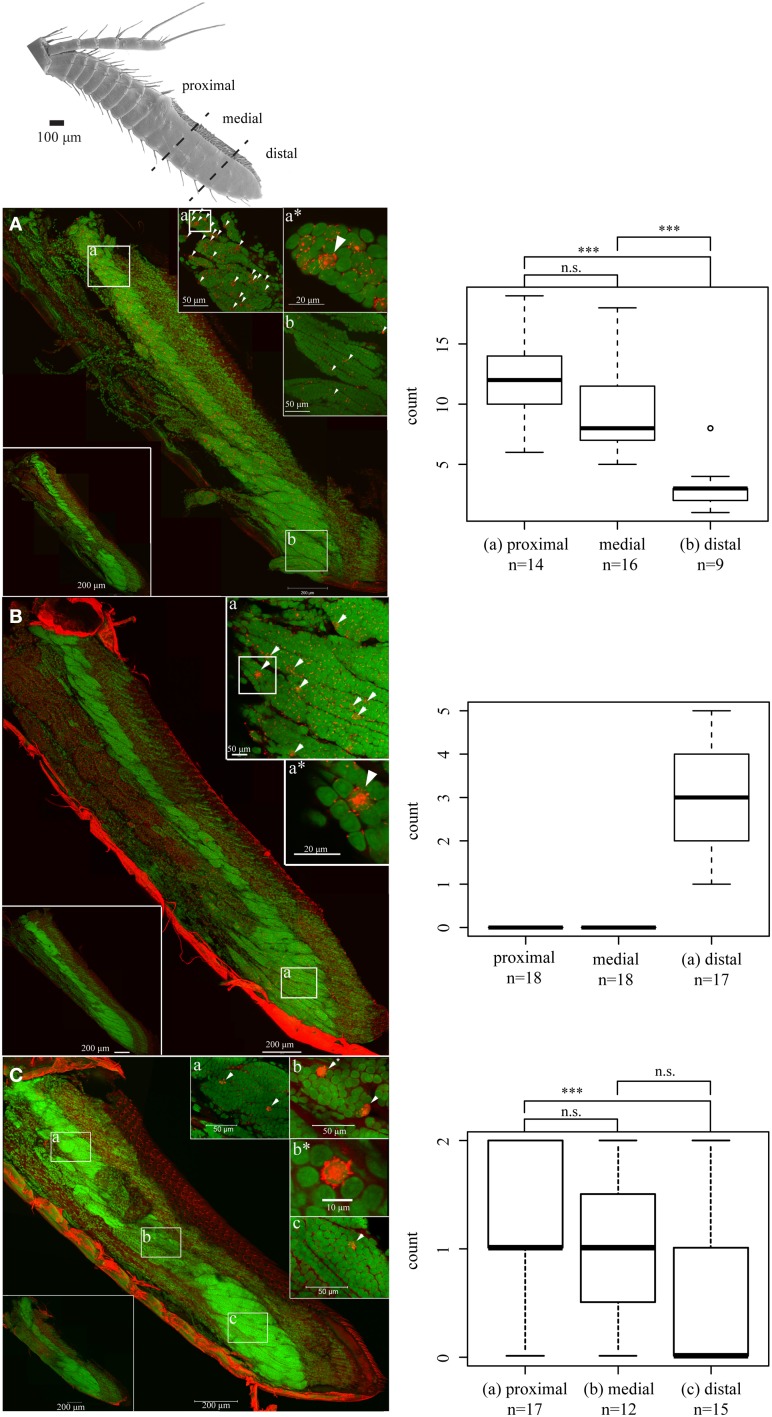
**LSM scan of whole mount fluorescence *in situ* hybridization**. Nuclear stain: SYTOX blue, Small letters refer to the respective region of higher magnification scan, asterisks to enlarged details; signals indicated by arrowheads, Boxplot: Signal count statistics and distribution significance of the respective regions (One-Way ANOVA and Tukey's test), ^***^p < 0.001, **(A)** IR1, **(B)** IR6, **(C)** IR26.

To assess the potential role of our IR candidates that belong neither to the antennal nor to the divergent IRs, we designed RNA probes to verify expression of the respective receptors in OSN cell bodies. Antisense probes of three IRs (IR1, IR6, and IR26) produced specific signals in distinct cells of several OSN cell body clusters. Sense control probes never produced signals in any cells of the antennules (Figure 4, small captions to the lower left in A–C). To evaluate the distribution of IR expressing cells, we counted and compared signals in OSN clusters in the basal, medial and distal antennular region. IR1 was expressed in all OSN clusters seen in the aesthetasc bearing antennular region (Figure 4A). In the elongated cell body complexes of the distal third an average of three IR1-expressing cells could be found (Figure 4Ab), significantly less than in both other regions (average: 12 and 9 cells, respectively) (Figure 4Aa). IR6-expressing cells were only detected in the distal third, with numbers varying between one and five cells per cluster (Figure 4B). IR26 was found only in OSN cell body complexes of the proximal two third with 1–2 cells per cluster. No IR26 expressing cells could be detected in the distal third (Figure 4C).

### Electrophysiology

**Figure 5:**
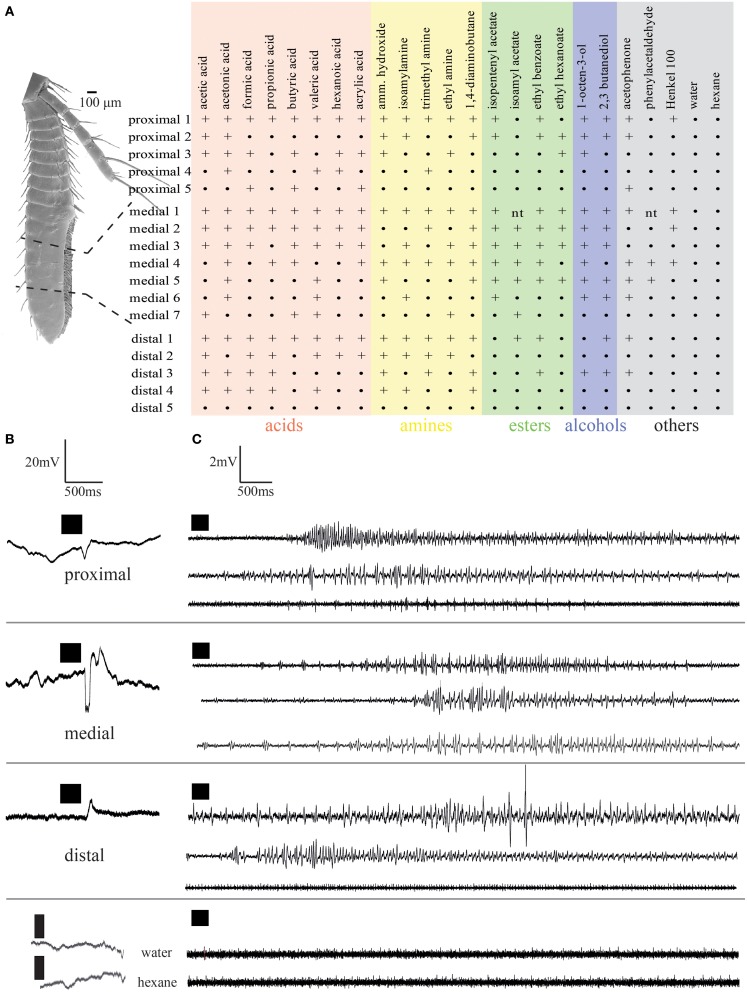
**Electrophysiological responses elicited by odor stimulation measured as changes of local field potentials (A,B) and changes in spike frequency (C). (A)** Response spectra of single aesthetascs in the respective antennular regions. Numbers refer to individual preparations. +, Response;•, no response; nt, not tested; **(B,C)**, responses to stimulation by ammonium hydroxide recorded as **(B)** local field potentials, **(C)** single sensillum recordings. Each trace represents an individual preparation. Time of odor application indicated by black squares.

**Figure 6:**
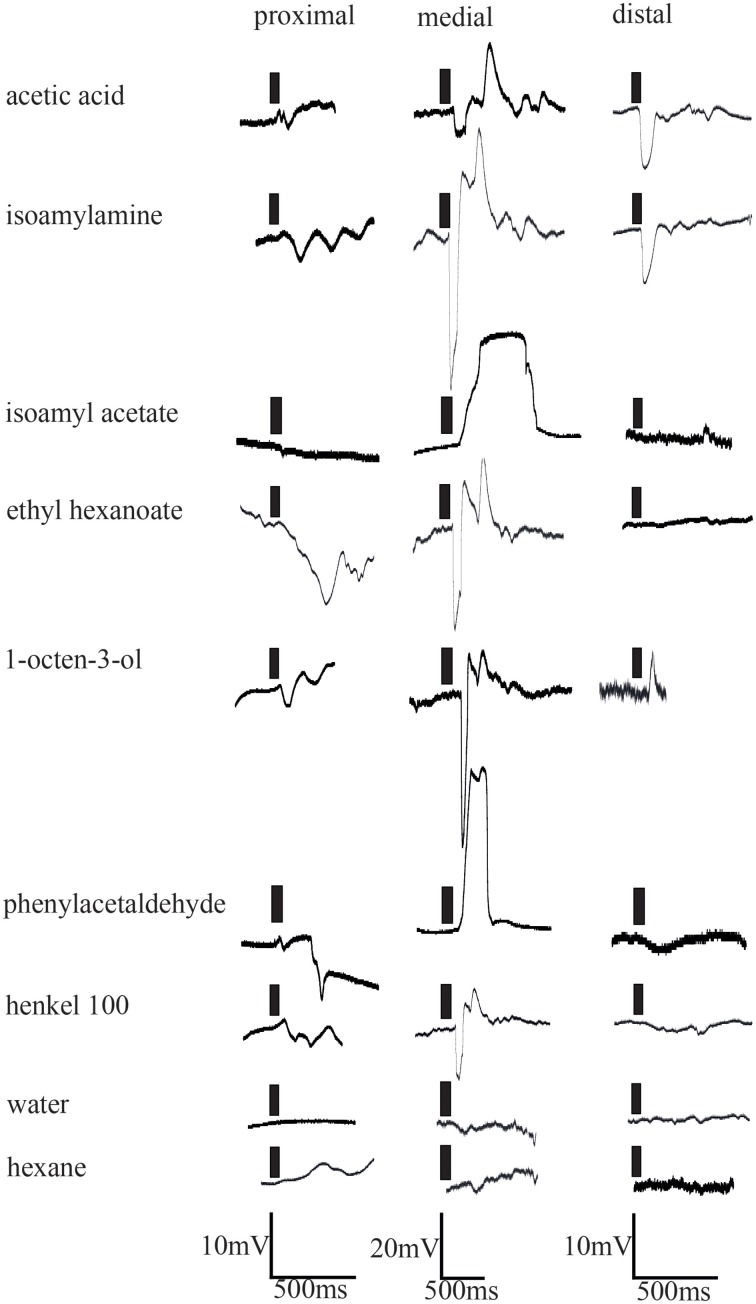
**Electrophysiological responses elicited by odor stimulation measured as changes of local field potentials of representative aesthetascs in the three aesthetasc field regions**. Time of odor application indicated by black bars.

The results of *in situ* hybridization indicated the distribution of cells expressing individual IRs to depend on the respective antennular region. To see if physiological responses between individual aesthetascs and between the regions were different, extracellular recordings of single aesthetascs and LFP recordings were taken from aesthetascs in the three regions as stated in the method. In extracellular recordings from single aesthetascs, odors stimulation of antennules evoked both a change in spike rate and a deflection in the LFP (Figure 5). All odors tested are listed in the upper panel of Figure 5A. LFP recordings both within and between the three regions exhibited different ligand spectra of individual aesthetascs (Figures 5A, 6). Additionally, the spiking patterns of individual neurons differed after stimulation with ammonium hydroxide (Figure 5C). Stimulation with solvents did not result in any response.

## Discussion

This study shows for the first time that the terrestrial hermit crab *Coenobita clypeatus* expresses a non-insect subclass of Ionotropic Receptors in its OSNs.

The current expansion of our earlier transcriptome analysis confirmed the representation of genes in the tissue, allowing annotation by BLAST for only 25% of the contigs (for comparison see Groh et al., 2014). Nevertheless, the total contig count and average contig length were substantially higher than in our previous study, likely due to increased read number. For general comparison with our earlier data we again performed BLAST2GO based annotation with parameters identical to our earlier study. While the number of contigs assigned each term was increased as expected, the proportional distribution of contigs to terms on a given level was highly similar. With few exceptions, the contig proportions assigned to a term on any given level was smaller than 2%, though the total number of assigned contigs was three to tenfold higher (see representative data of the category “Biological Process” in Supplementary Table 3, Groh et al., 2014).

As the antennules are the main olfactory organ of crustaceans we searched for representatives of genes involved in olfaction and olfactory processing in the hermit crabs antennules. Since the presence of IR25a has been documented in almost all or all OSN cell bodies of lobster antennules (Hollins et al., 2003; Tadesse et al., 2011; Corey et al., 2013) and studies of crustacean antennules point to IRs as only known putative chemosensory receptors expressed (Corey et al., 2013; Groh et al., 2014) we focused on candidate IRs. According to previous results (Corey et al., 2013; Groh et al., 2014), members of the so called divergent IR subgroup are expressed in crustacean antennules, while in insects only antennal IRs are (Benton et al., 2009; Croset et al., 2010). Our study demonstrates that the IR co-receptor IR25a protein is, in a similar fashion, expressed in all *Coenobita clypeatus* OSN cell bodies and is further present along the OSN dendrites to the aesthetascs. Therefore it is highly likely, that IRs form the basis of olfaction in terrestrial hermit crabs. Homology searches based on known crustacean IRs led to identification of 22 novel divergent IR candidates expressed in the *C. clypeatus* antennules, extending the total number of antennaly expressed IRs to 29. Our whole mount *in situ* hybridization experiments not only visualized the expression of three IRs in the OSN cell bodies of *C. clypeatus* but uncovered unequal distribution patterns along the last antennular section. Based on olfactory responses of isolated annuli it has been assumed that crustacean antennules are compound noses with two unresponsive regions; the proximal proliferation zone and the distal senescence zone (Steullet et al., 2000). However, successful aesthetasc recordings from all parts of the hermit crab aesthetasc field, including the proximal region and the tip, demonstrate functional detection of olfactory stimuli in all regions of the organ. The ligand spectra of single aesthetascs, however, differ within one region and between the three regions, with no obvious compound-specific pattern connected to the distribution of individual IRs. Likely due to a higher sensitivity of LFP recordings compared to electroantennographic measurements, we could detect responses to a number of esters, a ketone and an aldehyde; compounds found inactive in an earlier study of *C. clypeatus* (Krång et al., 2012). Individual odors did either depolarize or hyperpolarize individual dendrites, a fact that was already investigated in lobster OSNs and was linked to excitation versus inhibition of the respective neuron (Michel et al., 1991). Whether this principle applies to *Coenobita* OSNs appears likely but remains to be tested. Another important point pending further investigations is the ligand specificity of individual IRs and the combinatorial pattern of IRs expressed in one neuron. Together, this will lead to a better understanding of the peripheral olfactory coding possibilities in the hermit crab antennulae. Another open question is the transfer of odors from the air to the receptor exposing dendrite. Water soluble odors are believed to dissolve in the mucus covering the aesthetascs and diffuse through the cuticle into the receptor lymph space where it contacts the dendrite (Derby et al., 1997; DeForest and Reidenbach, 2012; Tuchina et al., 2014). In insects, water insoluble compounds are transferred by carrier proteins, the odorant binding proteins (OPB) (Pelosi et al., 2006). As no related proteins have been found in crustaceans antennules it still has to be determined how water insoluble odorants like acetophenone and phenylacetaldehyde reach their receptor. It has been suspected, that enzymes like the CUB domain containing serine protease, produced and secreted in aesthetasc associated glands of lobsters and also found in *C. clypeatus* antennal tegumental glands, might participate in olfaction by breaking down compounds for easier diffusion (Levine and Harrison, 2001; Tuchina et al., 2014). Whether an enzymatic activity or a yet unknown class of carrier proteins facilitates odor uptake remains to be investigated. However, our study identifies the chemosensory receptors expressed in hermit crab OSNs to be a subgroup of IRs, distinct from olfactory IRs of insects and with a broader ligand spectrum than those of insects (Silbering et al., 2011).

## Author contributions

Bioinformatics and immunohistochemistry experiments were carried out by Katrin C. Groh-Lunow. Merid N. Getahun performed the electrophysiology. *In situ* hybridization experiments were carried out by Katrin C. Groh-Lunow and Bill S. Hansson and Ewald Grosse-Wilde participated in study design, coordination and drafting of the manuscript. Katrin C. Groh-Lunow wrote the paper. Reagents and analytic tools were provided by Bill S. Hansson. All authors read and approve the final manuscript.

### Conflict of interest statement

The authors declare that the research was conducted in the absence of any commercial or financial relationships that could be construed as a potential conflict of interest.
