# Supplementary material for: Expression of ionotropic receptors in terrestrial hermit crab's olfactory sensory neurons
Source: Front Cell Neurosci. 2015 Feb 2;8:448. doi: 10.3389/fncel.2014.00448 (PMC4313712; doi:10.3389/fncel.2014.00448)
Supplement: Supplementary file 1 [file Table1.PDF]

CCcutoff10lvl2

GO ID	Term	#Seqs	percent
GO:0005623	cell	6231	31.92
GO:0043226	organelle	4873	24.97
GO:0016020	membrane	3225	16.52
GO:0032991	macromolecular complex	2898	14.85
GO:0031974	membrane-enclosed lumen	1005	5.15
GO:0005576	extracellular region	563	2.88
GO:0030054	cell junction	338	1.73
GO:0045202	synapse	231	1.18
GO:0031012	extracellular matrix	72	0.37
GO:0019012	virion	61	0.31
GO:0009295	nucleoid	22	0.11

CCcutoff10lvl3

GO ID	Term	#Seqs	percent
GO:0044464	cell part	6230	29.04
GO:0043227	membrane-bounded organelle	4245	19.78
GO:0044422	organelle part	3097	14.43
GO:0043234	protein complex	2326	10.84
GO:0044425	membrane part	1854	8.64
GO:0043228	non-membrane-bounded organelle	1807	8.42
GO:0031982	vesicle	562	2.62
GO:0044421	extracellular region part	445	2.07
GO:0005911	cell-cell junction	242	1.13
GO:0044456	synapse part	162	0.76
GO:0032993	protein-DNA complex	109	0.51
GO:0070161	anchoring junction	97	0.45
GO:0044423	virion part	60	0.28
GO:0048475	coated membrane	58	0.27
GO:0030055	cell-substrate junction	48	0.22
GO:0031594	neuromuscular junction	46	0.21
GO:0019867	outer membrane	46	0.21
GO:0044420	extracellular matrix part	22	0.10

BPcutoff10lvl2

GO ID	Term	#Seqs	percent
GO:0009987	cellular process	7647	18.07
GO:0008152	metabolic process	6983	16.50
GO:0044699	single-organism process	6708	15.85
GO:0065007	biological regulation	3777	8.92
GO:0050896	response to stimulus	3066	7.24
GO:0051179	localization	2551	6.03
GO:0071840	cellular component organization or biogenesis	2391	5.65
GO:0032501	multicellular organismal process	2179	5.15
GO:0032502	developmental process	2088	4.93
GO:0023052	signaling	1926	4.55
GO:0051704	multi-organism process	788	1.86
GO:0000003	reproduction	578	1.37
GO:0002376	immune system process	496	1.17
GO:0040011	locomotion	487	1.15
GO:0040007	growth	350	0.83
GO:0022610	biological adhesion	234	0.55
GO:0048511	rhythmic process	73	0.17

GO ID	Term	#Seqs	percent
GO:0071704	organic substance metabolic process	5943	8.94
GO:0044238	primary metabolic process	5731	8.62
GO:0044763	single-organism cellular process	5727	8.62
GO:0044237	cellular metabolic process	5656	8.51
GO:0006807	nitrogen compound metabolic process	3993	6.01
GO:0044710	single-organism metabolic process	3649	5.49
GO:0050789	regulation of biological process	3544	5.33
GO:0009058	biosynthetic process	2804	4.22
GO:0051234	establishment of localization	2285	3.44
GO:0051716	cellular response to stimulus	2201	3.31
GO:0016043	cellular component organization	2136	3.21
GO:0044707	single-multicellular organism process	2077	3.13
GO:0044767	single-organism developmental process	2071	3.12
GO:0044700	single organism signaling	1926	2.90
GO:0048856	anatomical structure development	1904	2.86
GO:0009056	catabolic process	1557	2.34
GO:0006950	response to stress	1248	1.88
GO:0044085	cellular component biogenesis	1163	1.75
GO:0042221	response to chemical	1100	1.66
GO:0065008	regulation of biological quality	996	1.50
GO:0051641	cellular localization	993	1.49
GO:0033036	macromolecule localization	852	1.28
GO:0009605	response to external stimulus	683	1.03
GO:0065009	regulation of molecular function	641	0.96
GO:0009628	response to abiotic stimulus	576	0.87
GO:0022414	reproductive process	514	0.77
GO:0016265	death	482	0.73
GO:0032504	multicellular organism reproduction	408	0.61
GO:0048610	cellular process involved in reproduction	368	0.55
GO:0008283	cell proliferation	347	0.52
GO:0007610	behavior	320	0.48
GO:0009719	response to endogenous stimulus	319	0.48
GO:0009607	response to biotic stimulus	307	0.46
GO:0006955	immune response	297	0.45
GO:0051674	localization of cell	246	0.37
GO:0007155	cell adhesion	234	0.35
GO:1902578	single-organism localization	184	0.28
GO:0032259	methylation	172	0.26
GO:0044419	interspecies interaction between organisms	110	0.17
GO:0044764	multi-organism cellular process	107	0.16
GO:0002252	immune effector process	99	0.15
GO:0015976	carbon utilization	85	0.13
GO:0051606	detection of stimulus	73	0.11
GO:0007623	circadian rhythm	61	0.09
GO:0044706	multi-multicellular organism process	60	0.09
GO:0043473	pigmentation	53	0.08
GO:0071554	cell wall organization or biogenesis	43	0.06
GO:0002440	production of molecular mediator of immune response	39	0.06
GO:0019882	antigen processing and presentation	34	0.05

GO:0044033	multi-organism metabolic process	31	0.05
GO:0060361	flight	14	0.02

MFcutoff10lvl2

GO ID	Term	#Seqs	percent
GO:0005488	binding	7595	46.83
GO:0003824	catalytic activity	5499	33.90
GO:0005215	transporter activity	928	5.72
GO:0060089	molecular transducer activity	424	2.61
GO:0005198	structural molecule activity	395	2.44
GO:0004872	receptor activity	372	2.29
GO:0001071	nucleic acid binding transcription factor activity	280	1.73
GO:0030234	enzyme regulator activity	250	1.54
GO:0009055	electron carrier activity	153	0.94
GO:0000988	protein binding transcription factor activity	142	0.88
GO:0005085	guanyl-nucleotide exchange factor activity	101	0.62
GO:0016209	antioxidant activity	68	0.42
GO:0016247	channel regulator activity	12	0.07

MFcutoff10lvl3

GO ID	Term	#Seqs	percent
GO:0097159	organic cyclic compound binding	4008	14.18
GO:1901363	heterocyclic compound binding	3999	14.15
GO:0043167	ion binding	3574	12.65
GO:0005515	protein binding	3411	12.07
GO:0016787	hydrolase activity	2114	7.48
GO:0016740	transferase activity	1979	7.00
GO:0036094	small molecule binding	1945	6.88
GO:0097367	carbohydrate derivative binding	1412	5.00
GO:0016491	oxidoreductase activity	919	3.25
GO:0022857	transmembrane transporter activity	748	2.65
GO:0022892	substrate-specific transporter activity	727	2.57
GO:0004871	signal transducer activity	424	1.50
GO:0048037	cofactor binding	325	1.15
GO:0016874	ligase activity	300	1.06
GO:0003700	sequence-specific DNA binding transcription factor activity	280	0.99
GO:0003735	structural constituent of ribosome	214	0.76
GO:0008289	lipid binding	181	0.64
GO:0016829	lyase activity	181	0.64
GO:0016853	isomerase activity	169	0.60
GO:0000989	transcription factor binding transcription factor activity	139	0.49
GO:0003682	chromatin binding	135	0.48
GO:0008047	enzyme activator activity	107	0.38
GO:0004857	enzyme inhibitor activity	89	0.31
GO:0051540	metal cluster binding	88	0.31
GO:0030246	carbohydrate binding	85	0.30
GO:0060589	nucleoside-triphosphatase regulator activity	85	0.30
GO:0061134	peptidase regulator activity	62	0.22
GO:0005088	Ras guanyl-nucleotide exchange factor activity	59	0.21
GO:0005200	structural constituent of cytoskeleton	59	0.21
GO:0019207	kinase regulator activity	40	0.14
GO:0043021	ribonucleoprotein complex binding	37	0.13
GO:0033218	amide binding	32	0.11
GO:1901681	sulfur compound binding	31	0.11
GO:1901476	carbohydrate transporter activity	29	0.10
GO:0060090	binding, bridging	28	0.10
GO:0008144	drug binding	28	0.10
GO:0008307	structural constituent of muscle	28	0.10
GO:1901505	carbohydrate derivative transporter activity	21	0.07
GO:0005326	neurotransmitter transporter activity	21	0.07
GO:0009975	cyclase activity	18	0.06
GO:0019208	phosphatase regulator activity	18	0.06
GO:0051184	cofactor transporter activity	15	0.05
GO:0042302	structural constituent of cuticle	15	0.05
GO:0003823	antigen binding	14	0.05
GO:0019239	deaminase activity	14	0.05
GO:0051183	vitamin transporter activity	14	0.05
GO:0032451	demethylase activity	12	0.04
GO:0005201	extracellular matrix structural constituent	12	0.04
GO:0090484	drug transporter activity	11	0.04
