# Supplementary material for: Expression of ionotropic receptors in terrestrial hermit crab's olfactory sensory neurons
Source: Front Cell Neurosci. 2015 Feb 2;8:448. doi: 10.3389/fncel.2014.00448 (PMC4313712; doi:10.3389/fncel.2014.00448)
Supplement: Supplementary file 2 [file Table2.PDF]

GPCR signaling

Seq. Name	Seq. Description	Seq. Lengtł	#Hits	min. eValue	mean Simil	#GOs
Contig_9514	tachykinin-like peptides receptor 99d-like	2165	20	1.26E-139	74.55%	3
Contig_70368	rac serine threonine-protein kinase-like isoform 1	398	20	1.97E-58	79.15%	56
Contig_66414	inositol -trisphosphate receptor type 1 isoform 2	402	20	7.00E-47	76.60%	36
Contig_63563	guanine nucleotide-binding protein subunit alpha-11	256	20	3.36E-21	67.35%	15
Contig_63173	breast cancer anti-estrogen resistance protein 1-like	716	20	9.40E-71	63.30%	17
Contig_62858	frizzled-8	312	20	4.56E-43	81.70%	7
Contig_60748	inositol -trisphosphate receptor type 1-like	348	20	1.87E-35	69.35%	37
Contig_59969	metabotropic glutamate receptor	296	20	1.49E-07	76.85%	5
Contig_59278	calcitonin receptor	336	20	4.38E-44	67.45%	3
Contig_58014	5-hydroxytryptamine receptor 2a	1032	20	1.39E-53	88.00%	4
Contig_57812	5-hydroxytryptamine receptor 2b	254	20	1.19E-36	79.55%	4
Contig_56143	glutathione synthetase	341	20	1.70E-30	71.25%	19
Contig_54444	octopamine receptor beta-3r-like	248	20	5.96E-26	80.00%	5
Contig_5148	guanine nucleotide-binding protein subunit gamma-e-like	850	20	6.70E-35	94.40%	8
Contig_49713	leucine-rich transmembrane protein	253	20	2.34E-15	61.15%	2
Contig_4913	sex peptide receptor	549	20	1.32E-15	79.20%	9
Contig_4648	g protein alpha subunit	4065	20	2.56E-97	98.05%	14
Contig_44596	histamine h1 receptor	329	20	7.09E-28	67.00%	2
Contig_42665	guanine nucleotide-binding protein subunit beta-5-like	324	20	1.33E-43	87.15%	6
Contig_41748	guanine nucleotide binding protein (g protein) alpha 14	370	20	1.96E-26	69.70%	4
Contig_4013	regulator of g protein signaling	1086	20	6.03E-170	77.25%	5
Contig_39270	regulator of g-protein signaling 7-like	1125	20	2.57E-134	87.40%	5
Contig_38998	latrophilin cirl-like isoform 1	399	20	9.76E-15	71.05%	4
Contig_29010	protocadherin-like wing polarity protein stan-like	341	20	6.18E-40	75.90%	5
Contig_28204	g-protein coupled receptor grl101-like	293	20	1.39E-23	68.10%	3
Contig_2709	gtp-binding protein alpha gna	3639	20	2.65E-100	98.45%	5
Contig_2366	guanine nucleotide-binding protein g subunit alpha-like	1799	20	0	91.50%	6
Contig_20793	rho guanine nucleotide exchange factor 12	1056	20	1.14E-104	68.40%	11
Contig_20191	guanine nucleotide-binding protein g subunit alpha-like	3320	20	0	92.40%	5
Contig_19255	regulator of g protein signaling	272	19	4.07E-11	77.68%	7
Contig_17240	regulator of g-protein signaling 2	419	20	7.28E-23	73.85%	8
Contig_17228	heterotrimeric gtp-binding protein alpha subunit g-alpha-q	847	20	9.35E-98	94.10%	5
Contig_16804	tyramine receptor	2374	20	0	69.75%	3
Contig_13983	guanine nucleotide-binding protein subunit gamma-1-like	2033	20	6.45E-23	85.60%	4
Contig_13313	rho guanine nucleotide exchange factor 12	526	20	5.53E-21	71.75%	16
Contig_11124	guanine nucleotide-binding protein g subunit alpha	720	20	2.06E-142	94.80%	14
