# Supplementary material for: Expression of ionotropic receptors in terrestrial hermit crab's olfactory sensory neurons
Source: Front Cell Neurosci. 2015 Feb 2;8:448. doi: 10.3389/fncel.2014.00448 (PMC4313712; doi:10.3389/fncel.2014.00448)
Supplement: Supplementary file 3 [file Table3.PDF]

BPlvl2

GO.ID	Term	Groh et al. 2014		new data		
		SeqsCcly	PercentCcly	SeqsCcly	PercentCcly	Difference
GO:0022610	biological adhesion	49	0.54	234	0.55	0.01
GO:0065007	biological regulation	773	8.57	3777	8.92	0.35
GO:0071840	cellular component organization or biogenesis	634	7.03	2391	5.65	1.38
GO:0009987	cellular process	1437	15.93	7647	18.07	2.14
GO:0032502	developmental process	554	6.14	2088	4.93	1.21
GO:0040007	growth	101	1.12	350	0.83	0.29
GO:0002376	immune system process	166	1.84	496	1.17	0.67
GO:0051179	localization	525	5.82	2551	6.03	0.21
GO:0040011	locomotion	120	1.33	487	1.15	0.18
GO:0008152	metabolic process	1386	15.36	6983	16.50	1.14
GO:0032501	multicellular organismal process	582	6.45	2179	5.15	1.30
GO:0051704	multi-organism process	268	2.97	788	1.86	1.11
GO:0000003	reproduction	180	1.99	578	1.37	0.62
GO:0050896	response to stimulus	633	7.02	3066	7.24	0.22
GO:0048511	rhythmic process	20	0.22	73	0.17	0.05
GO:0023052	signaling	369	4.09	1926	4.55	0.46
GO:0044699	single-organism process	1226	13.59	6708	15.85	2.26

		Groh et al. 2014		new data		
GO.ID	Term	SeqsCcly	PercentCcly	SeqsCcly	PercentCcly	Difference
GO:0048856	anatomical structure development	507	3.26	1904	2.86	0.40
GO:0019882	antigen processing and presentation	52	0.33	34	0.05	0.28
GO:0007610	behavior	70	0.45	320	0.48	0.03
GO:0009058	biosynthetic process	719	4.62	2804	4.22	0.40
GO:0015976	carbon utilization	13	0.08	85	0.13	0.05
GO:0009056	catabolic process	389	2.5	1557	2.34	0.16
GO:0007155	cell adhesion	49	0.32	234	0.35	0.03
GO:0008283	cell proliferation	119	0.77	347	0.52	0.25
GO:0071554	cell wall organization or biogenesis	9	0.06	43	0.06	0.00
GO:0044085	cellular component biogenesis	365	2.35	1163	1.75	0.60
GO:0016043	cellular component organization	567	3.65	2136	3.21	0.44
GO:0051641	cellular localization	253	1.63	993	1.49	0.14
GO:0044237	cellular metabolic process	1208	7.77	5656	8.51	0.74
GO:0048610	cellular process involved in reproduction	117	0.75	368	0.55	0.20
GO:0051716	cellular response to stimulus	446	2.87	2201	3.31	0.44
GO:0007623	circadian rhythm	14	0.09	61	0.09	0.00
GO:0016265	death	176	1.13	482	0.73	0.40
GO:0051606	detection of stimulus	12	0.08	73	0.11	0.03
GO:0051234	establishment of localization	477	3.07	2285	3.44	0.37
GO:0060361	flight	1	0.01	14	0.02	0.01
GO:0002252	immune effector process	27	0.17	99	0.15	0.02
GO:0006955	immune response	78	0.5	297	0.45	0.05
GO:0044419	interspecies interaction between organisms	120	0.77	110	0.17	0.60
GO:0051674	localization of cell	58	0.37	246	0.37	0.00
GO:0033036	macromolecule localization	230	1.48	852	1.28	0.20
GO:0032259	methylation	51	0.33	172	0.26	0.07
GO:0032504	multicellular organism reproduction	113	0.73	408	0.61	0.12
GO:0044706	multi-multicellular organism process	21	0.14	60	0.09	0.05
GO:0044764	multi-organism cellular process	115	0.74	107	0.16	0.58
GO:0044033	multi-organism metabolic process	43	0.28	31	0.05	0.23
GO:0006807	nitrogen compound metabolic process	909	5.85	3993	6.01	0.16
GO:0071704	organic substance metabolic process	1260	8.1	5943	8.94	0.84
GO:0043473	pigmentation	20	0.13	53	0.08	0.05
GO:0044238	primary metabolic process	1222	7.86	5731	8.62	0.76
GO:0002440	production of molecular mediator of immune response	6	0.04	39	0.06	0.02
GO:0050789	regulation of biological process	724	4.66	3544	5.33	0.67
GO:0065008	regulation of biological quality	250	1.61	996	1.50	0.11
GO:0065009	regulation of molecular function	170	1.09	641	0.96	0.13
GO:0022414	reproductive process	145	0.93	514	0.77	0.16
GO:0009628	response to abiotic stimulus	89	0.57	576	0.87	0.30
GO:0009607	response to biotic stimulus	60	0.39	307	0.46	0.07
GO:0042221	response to chemical	290	1.87	1100	1.66	0.21
GO:0009719	response to endogenous stimulus	92	0.59	319	0.48	0.11
GO:0009605	response to external stimulus	140	0.9	683	1.03	0.13
GO:0006950	response to stress	334	2.15	1248	1.88	0.27
GO:0044700	single organism signaling	369	2.37	1926	2.90	0.53
GO:0044707	single-multicellular organism process	569	3.66	2077	3.13	0.53
GO:0044763	single-organism cellular process	1101	7.08	5727	8.62	1.54
GO:0044767	single-organism developmental process	551	3.54	2071	3.12	0.42
GO:1902578	single-organism localization	85	0.55	184	0.28	0.27
GO:0044710	single-organism metabolic process	759	4.88	3649	5.49	0.61
